# Genome-wide association study meta-analysis supports association between MUC1 and ectopic pregnancy

**DOI:** 10.1093/humrep/dead217

**Published:** 2023-10-24

**Authors:** Natàlia Pujol Gualdo, Estonian Biobank Research Team, Reedik Mägi, Triin Laisk

**Affiliations:** Estonian Genome Centre, Institute of Genomics, University of Tartu, Tartu, Estonia; Department of Obstetrics and Gynecology, Research Unit of Clinical Medicine, University of Oulu, Oulu, Finland; Estonian Genome Centre, Institute of Genomics, University of Tartu, Tartu, Estonia; Estonian Genome Centre, Institute of Genomics, University of Tartu, Tartu, Estonia; Estonian Genome Centre, Institute of Genomics, University of Tartu, Tartu, Estonia

**Keywords:** GWAS, ectopic pregnancy, MUC1, genome-wide association study, pregnancy complication

## Abstract

**STUDY QUESTION:**

Can we identify genetic variants associated with ectopic pregnancy by undertaking the first genome-wide association study (GWAS) leveraging two large-scale biobank initiatives?

**SUMMARY ANSWER:**

We identified two novel genome-wide significant associations with ectopic pregnancy, highlighting *MUC1* (mucin 1) as the most plausible affected gene.

**WHAT IS KNOWN ALREADY:**

Ectopic pregnancy is an important cause of maternal morbidity and mortality worldwide. Despite being a common early pregnancy complication, the genetic predisposition to this condition remains understudied and no large scale genetic studies have been performed so far.

**STUDY DESIGN, SIZE, DURATION:**

A GWAS meta-analysis including 7070 women with ectopic pregnancy and 248 810 controls from Estonian Biobank and the FinnGen study.

**PARTICIPANTS/MATERIALS, SETTING, METHODS:**

We identified ectopic pregnancy cases from national registers by ICD (International Classification of Disease) codes (ICD-10 O00), and all remaining women were considered controls. We carried out standard GWAS meta-analysis and additionally annotated GWAS signals, analysed co-localization with quantitative trait loci, estimated genetic correlations and identified associated phenotypes to characterize the genetic signals, as well as to analyse the genetic and phenotypic relationships with the condition.

**MAIN RESULTS AND THE ROLE OF CHANCE:**

We identified two genome-wide significant loci on chromosomes 1 (rs4971091, *P* = 5.32×10^−9^) and 10 (rs11598956, *P* = 2.41×10^−8^) potentially associated with ectopic pregnancy. Follow-up analyses propose *MUC1*, which codes for an epithelial glycoprotein with an important role in barrier function, as the most likely candidate gene for the association on chromosome 1. We also characterize the phenotypic and genetic correlations with other phenotypes, identifying a genetic correlation with smoking and diseases of the (genito)urinary and gastrointestinal system, and phenotypic correlations with various reproductive health diagnoses, reflecting the previously known epidemiological associations.

**LARGE SCALE DATA:**

The GWAS meta-analysis summary statistics are available from the GWAS Catalogue (GCST90272883).

**LIMITATIONS, REASONS FOR CAUTION:**

The main limitation is that the findings are based on European-based ancestry populations, with limited data on other populations, and we only captured maternal genomes. Additionally, further larger meta-analysis or independent studies are needed to validate these findings.

**WIDER IMPLICATIONS OF THE FINDINGS:**

This study encourages the use of large-scale genetic datasets to unravel genetic factors linked to ectopic pregnancy, which is difficult to study in experimental settings. Increased sample size might bring additional genetic factors associating with ectopic pregnancy and inform its heritability. Altogether, our results provide more insight into the biology of ectopic pregnancy and, accordingly, the biological processes governing embryo implantation.

**STUDY FUNDING/COMPETING INTEREST(S):**

N.P.G. was supported by MATER Marie Sklodowska-Curie which received funding from the European Union’s Horizon 2020 research and innovation program under grant agreement No. 813707. This study was funded by European Union through the European Regional Development Fund Project No. 2014-2020.4.01.15-0012 GENTRANSMED. Computations were performed in the High-Performance Computing Center of University of Tartu. The authors declare no competing interests.

## Introduction

An ectopic pregnancy is a pregnancy complication where the fertilized oocyte implants and grows outside the uterine cavity, mostly (95% of cases) in the Fallopian tube. Studies suggest that tubal ectopic pregnancy is a result of a combination of retention of the embryo within the Fallopian tube due to impaired embryo-tubal transport and alterations in the tubal environment allowing premature implantation (Shaw *et al.*, 2010). Ectopic pregnancy is one of the most common early pregnancy complications, affecting around 1–2% of all pregnancies, and is also the major cause of maternal mortality in the first trimester, accounting for ∼6% of all maternal deaths (Panelli *et al.*, 2015).

The symptoms of ectopic pregnancy include abdominal pain and/or vaginal bleeding, and it is diagnosed by hCG testing and ultrasound. Although in some cases an ectopic pregnancy miscarries on its own, usual treatment includes methotrexate or surgery.

Known risk factors of ectopic pregnancy include maternal age, smoking, tubal surgery, tubal adhesions or blockage due to pelvic inflammatory disease, and the use of assisted reproductive technologies. Having an ectopic pregnancy also increases the risks of having another one, with a recurrence rate of 5–25% (Panelli *et al.*, 2015). Women with a previous ectopic pregnancy are also at an increased risk of compromised future fertility, post-traumatic stress, depression, and anxiety (Lund Karhus *et al.*, 2013; Farren *et al.*, 2020).

It has been shown that daughters of mothers with ectopic pregnancy have a 50% higher risk of ectopic pregnancy compared to daughters of women without an ectopic pregnancy (Kårhus *et al.*, 2014), indicating a potentially heritable component to the condition. A recent study in mice suggested the gene *Adgrd1* might control oviductal fluid flow and embryo transit and thus be involved in ectopic pregnancy (Bianchi *et al.*, 2021). Similarly, ectopic pregnancies have been reported in individuals with primary ciliary dyskinesia, a condition with genetic origins (Blyth and Wellesley, 2008; Mirra *et al.*, 2017). However, no large-scale studies to explore the potential contribution from genetic factors in ectopic pregnancy have been conducted.

Here, we report a genome-wide association study (GWAS) meta-analysis in 7070 ectopic pregnancy cases and 248 810 controls. We identify two genome-wide significant signals and thus provide the first evidence of a genetic susceptibility component to ectopic pregnancy in humans.

## Materials and methods

### Study cohorts

Our analyses included a total of 7070 women with ectopic pregnancy and 248 810 female controls of European ancestry from two studies: summary level statistics from the FinnGen R7 data release (4526 cases and 124 547 female controls, prevalence 3.5%) for the phenotype O15_PREG_ECTOP, and individual level data from the Estonian Biobank (EstBB, 2544 cases and 124 263 female controls, prevalence 2%) (Supplementary Fig. S1). In the EstBB, cases were defined as women having ectopic pregnancy diagnosed by International Classification of Disease (ICD-10) diagnosis code O00. Controls were defined as women who did not have the respective ICD code. We ran an additional analysis in the EstBB identifying controls as women who have ever been pregnant or have ever delivered (obtained from questionnaire data or from ICD-codes: Z32—Pregnancy examination and test, O80-O84—Delivery), this analysis included 53 671 controls.

### Cohort-level analyses

The EstBB is a population-based biobank with over 200 000 participants (corresponding to 20% of the total Estonian population), currently including around 135 000 women. All biobank participants have signed a broad informed consent form and analyses were carried out under ethical approval 1.1-12/624 from the Estonian Committee on Bioethics and Human Research (Estonian Ministry of Social Affairs) and data release N05 from the EstBB. Individuals with ectopic pregnancy were identified using the ICD-10 code O00 (mean age at recruitment = 38.4 years, SD = 10.5), and all female biobank participants who did not have this diagnosis were considered as controls (mean age at recruitment = 44.9 years, SD = 16.3), which resulted in 2544 cases and 124 263 female controls (prevalence 2%). The age range of the selected controls was 49.7 ± 14.2 years. Information on ICD codes is obtained via regular linking with the National Health Insurance Fund and other relevant databases (Leitsalu *et al.*, 2015).

Details of EstBB genotyping procedure have been described previously (Laisk *et al.*, 2021; Pujol-Gualdo *et al.*, 2022; Koel *et al.*, 2023). Briefly, all EstBB participants were genotyped using Illumina GSAv1.0, GSAv2.0, and GSAv2.0_EST arrays at the Core Genotyping Lab of the Institute of Genomics, University of Tartu. Individuals were excluded from the analysis if their call-rate was <95% or if their sex defined by heterozygosity of X chromosomes did not match their sex in the phenotype data. Before imputation, variants were filtered by call-rate <95%, Hardy–Weinberg equilibrium (HWE) *P*-value <1e^−4^ (autosomal variants only), and minor allele frequency <1%. Same analyses were conducted for association analysis and imputation of chromosome X, except for the HWE filter, which was not applied. Pre-phasing was conducted using Eagle v2.3 software (Loh *et al.*, 2016) and imputation was done using Beagle v.28Sep18.793 (Browning and Browning, 2007). The population specific imputation reference of 2297 whole genome sequencing samples was used (Mitt *et al.*, 2017). Association analysis was carried out using REGENIE (v2.2.4) (Mbatchou *et al.*, 2021), which uses a mixed-model-based approach and is thus suitable for datasets containing relatives. Year of birth and 10 genetic principal components were used as covariates in Step I. Inclusion of genetic principal components as covariates controls for any undesired population stratification. By default variants with a minor allele count <5 were excluded. Analyses were run using an additive model (results for the independent GWAS studies are shown on Supplementary Fig. S2). In EstBB, variants with poor imputation quality (imputation INFO score < 0.4) were excluded from downstream association analysis.

For FinnGen, we used GWAS summary statistics from the R7 data release and thus did not have individual level data. In the FinnGen data, cases were defined as women having a respective ICD8 (631), ICD9 (633), or ICD10 (O00) code for ectopic pregnancy, while controls were women who did not have any of the respective codes (https://r7.risteys.finngen.fi/phenocode/O15_PREG_ECTOP). The summary statistics were downloaded as described here: https://www.finngen.fi/en/access_results. The FinnGen cohort and the relevant genotyping/data analysis details have been described in Kurki *et al.* (2023). Briefly, REGENIE 2.0.2 (Mbatchou *et al.*, 2021) was used for analysis, with age, 10 PCs, and genotyping batch as covariates. FinnGen summary statistics include variants with a minor allele count >5 and imputation INFO score 0.6.

### GWAS meta-analysis

We conducted an inverse variance weighted fixed-effects meta-analysis with genomic control using GWAMA (v2.1) (Mägi and Morris, 2010). The genomic inflation factors (lambda) of the individual study summary statistics were 1.037 (EstBB), and 1.036 (FinnGen). Genome-wide significance was set to *P* < 5 × 10^−8^ and for downstream analyses we included only variants present in both cohorts (n = 12 363 169 variants).

### Annotation of GWAS signals

We used FUMA (v1.4.0) (Watanabe *et al.*, 2017) to annotate the GWAS signals. FUMA is an online platform that performs annotation of GWAS signals using data from several databases. First, FUMA identifies lead single nucleotide polymorphisms (SNPs) (*P*-value <5 × 10^−8^ and linkage disequilibrium (LD) *r*^2^<0.1, based on 1000G European reference) and independent significant SNPs and each risk locus (*P*-value <5 × 10^−8^ and LD *r*^2^ < 0.6). Then FUMA identifies potential candidate SNPs that are in LD with any of the identified independent significant SNPs and annotates them via linking with several databases (ANNOVAR (Wang *et al.*, 2010), RegulomeDB (Boyle *et al.*, 2012), CADD (Kircher *et al.*, 2014) scores etc.), which gives information on their location, functional impact, and potential regulatory effects (Supplementary Table S1).

FUMA also links with the GWAS catalogue (https://www.ebi.ac.uk/gwas/) for previous associations between the identified candidate SNPs and studies in the GWAS catalog. The results of this look-up are presented in Supplementary Table S2.

### Co-localization analysis

We used HyPrColoc (Foley *et al.*, 2021), a co-localization method for identifying the overlap between our GWAS meta-analysis signals and cis-QTL (quantitative trait locus) signals from different tissues and cell types (expression QTLs, transcript QTLs, exon QTLs, and exon usage QTLs) available in the eQTL Catalogue (Kerimov *et al.*, 2021). We lifted the GWAS summary statistics over to hg38 build to match the eQTL Catalogue using binary liftOver tool (https://genome.sph.umich.edu/wiki/LiftOver#Binary_liftOver_tool).

For the genome-wide significant (*P* < 5 × 10^−8^) GWAS loci identified we extracted the ±500 kb of its top hit from QTL datasets and ran the co-localization analysis against eQTL Catalogue traits. For each eQTL Catalogue dataset, we included all the QTL features which shared at least 80% of tested variants with the variants present in our GWAS region. We used the default settings for HyPrColoc analyses and did not specify any sample overlap argument, because HyPrColoc paper (Foley *et al.*, 2021) demonstrates that assuming trait independence gives reasonable results. HyPrColoc outputs the posterior probability that genetic association signals for those traits are co-localizing (we considered two or more signals to co-localize if the posterior probability for a shared causal variant (PP4) was 0.8 or higher). All results with a PP4 > 0.8 can be found in Supplementary Table S3.

### Look-up of main signals in Biobank Japan

We looked up the association results for our European ancestry meta-analysis lead variants with summary statistics publicly available from Biobank Japan (Sakaue *et al.*, 2021), including 605 cases and 82 156 female controls (prevalence 0.7%) to compare association results in a different ancestry cohort in association with ectopic pregnancy. The Biobank Japan data were downloaded from https://pheweb.jp. Due to methodological limitations (the preferred multi-ancestry meta-analysis software MR-MEGA requires more than 3 cohorts for analysis), we were not able to include this dataset in a formal meta-analysis.

### Heritability analysis

We used LD-Score (LDSC) regression (Bulik-Sullivan *et al.*, 2015) and the HapMap3 reference panel to estimate the total SNP-based heritability (*h*^2^_SNP_) of the ectopic pregnancy meta-analysis. To convert observed heritability estimates to liability scale, we used a transformation tool which is suitable for low prevalence biobank phenotypes (https://medical-genomics-group.shinyapps.io/h2liab/) (Ojavee *et al.*, 2022) and we used a population prevalence equal to the study sample prevalence (2.76%).

### Genetic correlation analysis

We used the Complex Traits Genetics Virtual Lab (CTG-VL, https://genoma.io/) to calculate genetic correlations between our ectopic pregnancy meta-analysis and 1335 traits. We applied a multiple testing correction (false discovery rate, FDR < 5%) to determine statistical significance using the p.adjust function in R 3.6.3. Full results of the genetic correlation analysis are presented in Supplementary Table S4.

### Gene-based analysis and look-up of previous candidate genes

Gene-based testing was carried out with MAGMA v1.08 implemented in FUMA (de Leeuw *et al.*, 2015; Watanabe *et al.*, 2017). Additionally, we queried previous proposed candidate genes for ectopic pregnancy amongst our summary statistics results: *Adgrd1* (also known as *GPR113*) (Bianchi *et al.*, 2021), *VEGFA* (vascular endothelial growth factor A), *IL8* (interleukin 8), *IL6* (interleukin 6), *ESR1* (estrogen receptor 1), and *EGFR* (epidermal growth factor receptor) (Liu and Zhao, 2016). Results of this analysis can be found in Supplementary Table S5.

### Analysis of associated phenotypes in EstBB

Using the individual level data in the EstBB, we conducted an analysis to find ICD10 diagnosis codes associated with the O00 diagnosis. We tested the association between ectopic pregnancy (defined as ICD10 O00) and other ICD10 codes in a logistic regression framework, adjusting for age and 10 genetic PCs. Since the EstBB includes a large proportion of relatives and inclusion of relatives might inflate the association statistics, we excluded all first- and second-degree relatives (pi-hat cut-off value 0.2) in pairwise comparisons, keeping the index cases, if possible, to not lose in power. This resulted in 2370 cases and 42 970 controls for the analysis. Bonferroni correction was applied to select statistically significant associations (number of tested ICD main codes—2001, corrected *P*-value threshold—2.5 × 10^−5^). Results were visualized using the PheWAS library 0.99.5-4. All analyses were carried out in R 3.6.3. The results of this analysis are presented in Supplementary Table S6. We carried out the analysis using both unselected controls (all women) and selected controls (women who have been pregnant but have not had ectopic pregnancy). For clarity, we only present the results from the selected controls analysis.

### Regulatory and functional enrichment with GARFIELD

We tested enrichment of SNPs at functionally annotated regions using GARFIELD (Iotchkova *et al.*, 2019). Annotations were provided by the GARFIELD software (DNase I hypersensitivity hotspots, open chromatin peaks, transcription-factor footprints and formaldehyde-assisted isolation of regulatory elements, histone modifications, chromatin segmentation states, genic annotations, and transcription-factor binding sites). We used the GWAS meta-analysis summary statistics and applied GARFIELD to DNase I hypersensitivity hotspot annotation in 424 cell lines and primary cell types from ENCODE and Roadmap Epigenomics and derived enrichment estimates at trait-genotype association *P*-value thresholds of *P* < 5 × 10^−5^ and *P* < 5 × 10^−8^. Because our GWAS included only European individuals, we used the original files describing the allele frequencies and LD from the UK10K data provided in the GARFIELD distribution. We also used the annotation and distance to transcription start site files provided within GARFIELD. Results of GARFIELD enrichment analysis are shown in Supplementary Table S7.

## Results

### Genome-wide association study meta-analysis

The meta-analysis identified two loci for ectopic pregnancy, with three independent lead signals significantly associated with EP (*P* < 5 × 10^−8^). There was no evidence of inflation (λ = 1.0285) in the GWAS meta-analysis (LDSC intercept = 0.9706 (SE 0.0067)). The observed SNP heritability estimate was 0.0106 (SE 0.0019), which corresponds to a liability scale SNP heritability of 7.03% (SE 0.013).

**Figure 1.**
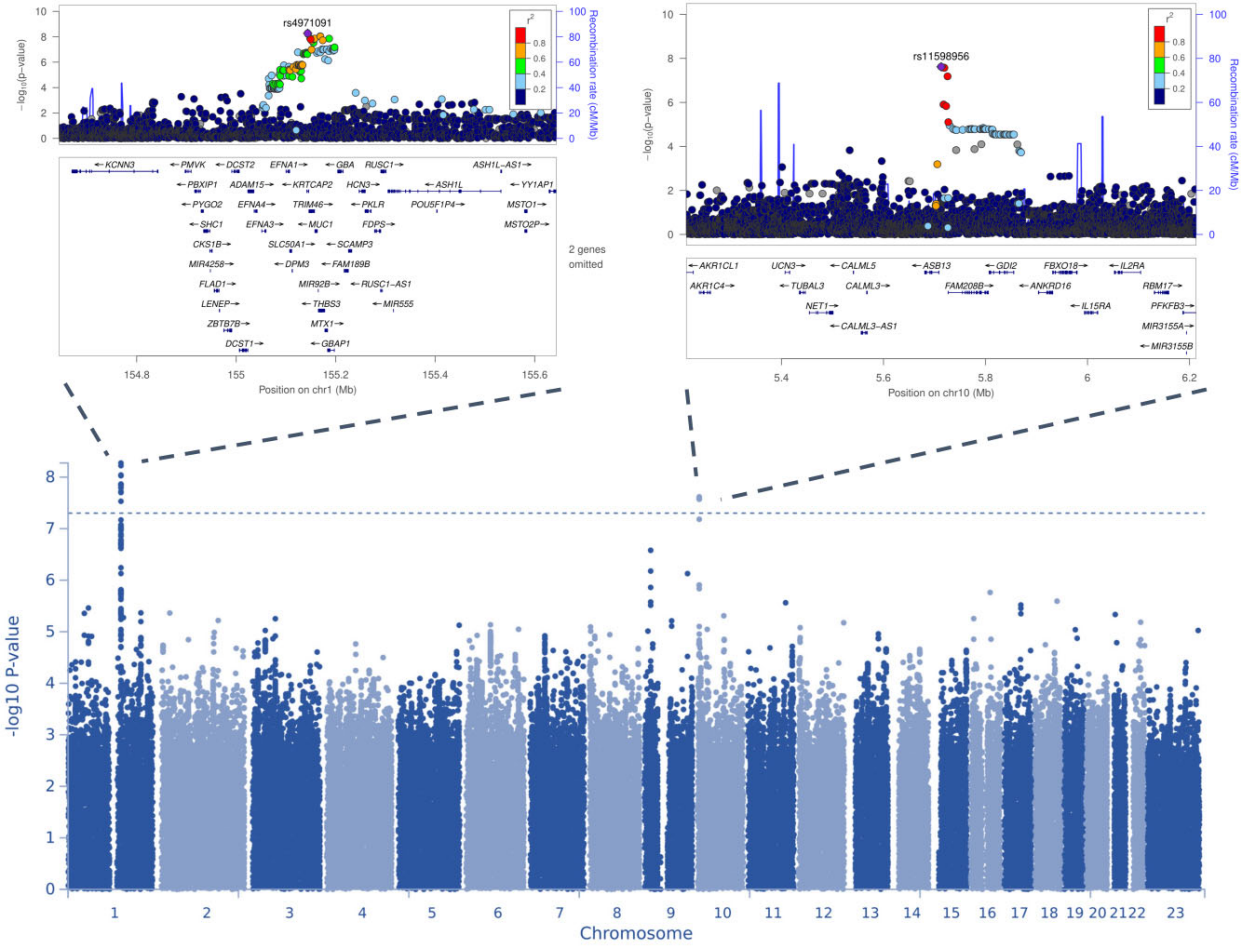
**Manhattan and locus zoom plots for genome-wide association study (GWAS) meta-analysis for ectopic pregnancy.** On the Manhattan plot, the *y* axis represents −log_10_(*P-*values) for association of variants with ectopic pregnancy. The horizontal dashed line represents the threshold for genome-wide significance (*P* < 5 × 10^−8^). Regional plots display the lead variants of genome-wide significant loci on chromosomes 1 (left) and 10 (right), respectively. The *x*-axis shows position on the chromosome in megabases (Mb), while the *y* axis represents −log_10_(*P-*values) for association (left) and recombination rate at each genomic position (right). The variant indicated in purple is the index variant, and other variants are colour coded according to their linkage disequilibrium (R-squared) with the index variant.

The first signal is a common variant (minor allele frequency 0.45) on chromosome 1 (lead signal rs4971091, *P* = 5.32 × 10^−9^), in the exon of a non-coding transcript *KRTCAP2* (Table 1). According to FUMA, there is another independent SNP rs1057941 (*P* = 1.38 × 10^−8^) in the GBAP1 pseudogene. As can be seen from Fig. 1, the signal is in a gene-dense region that also includes *MUC1* (mucin 1). Co-localization analysis showed our GWAS signal overlaps with QTL signals associated with both *MUC1* expression and specific transcripts (Supplementary Table S3). Further analysis of the association signal revealed that the second independent SNP in this region, rs1057941, is in LD with rs4072037 (*r*^2^_European = 0.52, *r*^2^_Finnish = 0.76, *r*^2^_Estonian = 0.69), a splice acceptor variant in the second exon of *MUC1* that in our meta-analysis has a *P*-value of 1.87 × 10^−7^ (C-allele odds ratio (OR) = 1.10 95% CI 1.06–1.13). According to the GWAS catalogue look-up, this region has previously been associated with gastric cancer and several biomarker levels such as liver enzyme levels (alanine transaminase, aspartate aminotransferase, gamma glutamyl transferase), red blood cell count, platelet count, haematocrit percentage, urate levels, urinary albumin-to-creatinine ratio, serum uric acid levels, cystatin C levels, serum phosphate levels, urea levels, magnesium levels, serum CC16 levels, and creatinine levels (Supplementary Table S2).

**Table 1:** Genome-wide significant lead variants in the two loci associated with ectopic pregnancy identified in the GWAS meta-analysis.

Locus	rsID (EA/NEA)	Variant annotation	chr:pos (hg19)	Meta-analysis P-value	EstBB *P*-value	FinnGen R7 *P*-value	OR (95% CI)	EAF	q-statistic in meta-analysis	q *P*-value	*i* ^2^ (%)
1q22	rs4971091 (G/T)	Non-coding transcript exon variant	1:155143768	5.32 × 10^−9^	3.0 × 10^−4^	2.27 × 10^−6^	1.11 (1.07–1.15)	0.55	0.01	0.90	0%
10p15.1	rs11598956 (A/G)	upstream gene variant	10:5713197	2.41 × 10^−8^	4.0 × 10^−2^	5.98 × 10^−8^	0.56 (0.46–0.68)	0.99	1.21	0.27	17.7%

Positions are according to build GRCh37.

EA, effect allele; NEA, non-effect allele; EAF, effect allele frequency; GWAS, genome-wide association study.

We identified a second signal on chromosome 10, where the lead SNP is rs11598956 (*P* = 2.41 × 10^−8^, minor allele frequency 0.6%, imputation INFO score >0.9 in both cohorts) between the *ASB13* and *TASOR2* genes. Of the candidate SNPs at this locus, rs74599685 in the intron of *ASB13* has a RegulomeDB score of 2b, which indicates evidence for a regulatory effect.

A sensitivity analysis carried out in the EstBB using controls who have been pregnant yielded similar results (data not shown), indicating that the use of unselected controls does not have an effect on the results. When comparing the two studies, none of the presented lead variants showed heterogeneity of effects between the two studies meta-analysed (Q-Cochran *P*-val = 0.90 and 0.27, *i*^2^ = 0% and 17%, for rs4971091 and rs11598956, respectively), which adds reliability to the described genetic associations and proves the homogeneity of cases definition criteria between studies.

Look-up of lead variants in the Biobank Japan dataset consisting of 605 cases and 82 156 female controls showed that although neither of the variants was statistically significantly associated with ectopic pregnancy, the effect directions were the same as in the European ancestry meta-analysis (rs4971091 G allele OR = 1.12, 95% CI 0.97–1.31; rs11598956 G allele OR = 1.07 95% CI 0.79–1.46).

### Regulatory and functional enrichment with GARFIELD

Using GARFIELD (Iotchkova *et al.*, 2019), we assessed for enrichment of our signals for DNase l hypersensitivity sites and chromatin accessibility peaks. For open chromatin peaks, we observed a high enrichment in hepatocytes. Overall we observed enrichment in a wide variety of cell types, including higher enrichment in blood, foetal muscle and foetal lung for GWAS loci associated with ectopic pregnancy (Supplementary Table S7).

### Gene-based analysis and look-up of previous candidate genes with MAGMA

Gene-based analysis implemented in MAGMA identified six associations significant after Bonferroni correction (0.05/18895 protein coding genes), all on chromosome 1—*RP11-201K10.3*, *THBS3*, *KRTCAP2*, *TRIM46*, *MTX1*, and *EFNA1*. Look-up of genes associated with ectopic pregnancy in previous literature (*ADGRD1* (*GPR113*), *VEGFA, IL8*, *IL6*, *ESR1*, and *EGFR*) revealed that none of them passed the Bonferroni correction threshold and only *GPR113* (also known as *ADGRD1*) and *ESR1* were nominally significant (*P* = 0.046 and *P* = 0.044, respectively). Full results are shown in Supplementary Table S5.

### Genetic correlation

**Figure 2.**
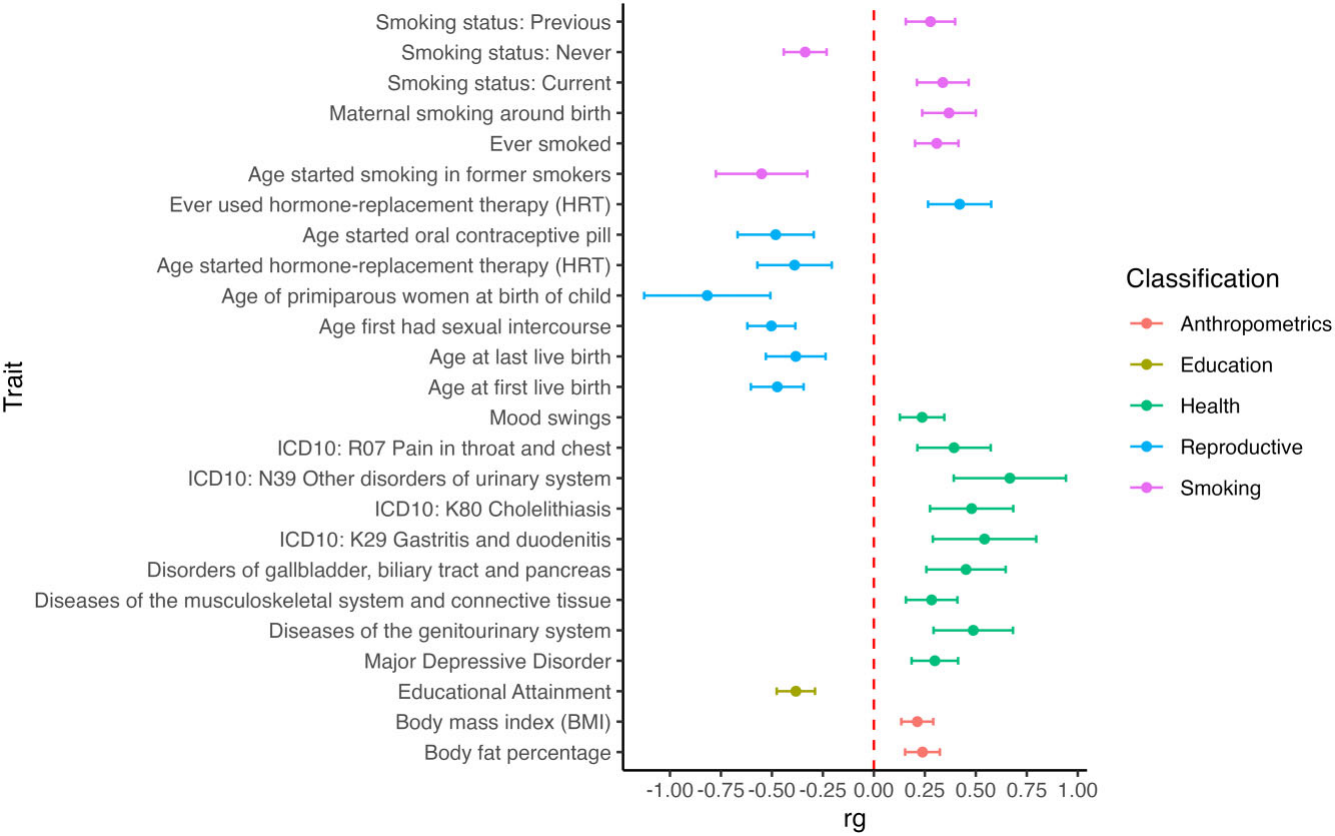
**Genetic correlations between ectopic pregnancy and anthropometric, educational, health-related, reproductive, and smoking phenotypes.** Points show the estimated genetic correlation (rg), which is presented as a dot and error bars indicate 95% confidence limits. Dotted red line indicates no genetic correlation.

We tested pairwise genetic correlations (*r*_g_) between ectopic pregnancy and 1335 other traits (Supplementary Table S4) available from the Complex Traits Genetics Virtual Lab (CTG-VL, https://genoma.io/). We found 93 significant (FDR < 0.05) genetic correlations with European-ancestry ectopic pregnancy meta-analysis. Selected genetic correlations with phenotypes related to smoking, overall and reproductive health, anthropometrics, and education are presented in Fig. 2. As expected, risk of ectopic pregnancy is associated with different smoking phenotypes. We also observed positive genetic correlations with diseases of the (genito)urinary and gastrointestinal system.

### Associated phenotypes

**Figure 3.**
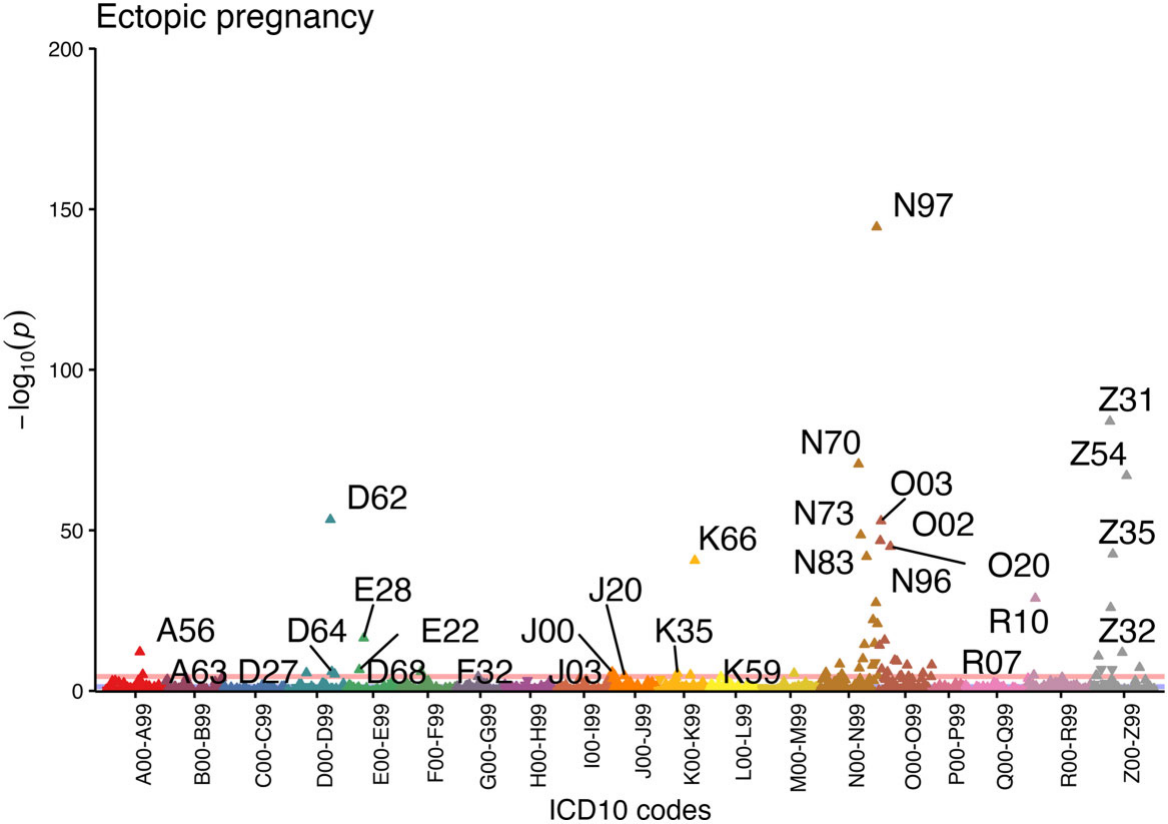
**Disease codes associated with a diagnosis of ectopic pregnancy (O00) in the Estonian Biobank**. Each triangle represents one ICD-10 (International Classification of Disease 10) maincode, while different colours correspond to different chapters. The direction of the triangle illustrates effect direction—upward pointing triangles show increased prevalence of the diagnosis code in ectopic pregnancy cases. The pink line shows the Bonferroni corrected threshold for statistical significance.

The significantly associated disease codes in the pheWAS analysis are consistent with what is known about the etiopathogenesis of the condition, validating the used phenotype definition. In our analysis, women with a diagnosis of ectopic pregnancy have significantly more diagnoses of female infertility (N97, OR = 3.4 (3.1–3.7)), procreative management (including IVF, Z31, OR = 3.3 (2.9–3.7)), salpingitis (N70, OR = 2.6 (2.3–2.9)), posthemorrhage anaemia (D62, OR = 6.3(5.0–7.9)), miscarriage (O02, OR = 2.3 (2.0–2.5); O03, OR = 2.6 (2.3–3.0)), other female pelvic inflammatory disease (N73, OR = 3.2 (2.7–3.7)), chlamydial infection (A56, OR = 1.6 (1.4–1.9)), bleeding in early pregnancy (O20, OR = 2.1 (1.9–2.3)), and abdominal pain (R10, OR = 1.6 (1.5–1.8)). In addition, we found increased odds of peritoneal disorders (K66, OR = 5.7 (4.4–7.3)), habitual abortion (N96, OR = 3.3 (2.7–4.1)), excessive, frequent, and irregular menstruation (N92, OR = 1.5 (1.4–1.7)), complications associated with artificial insemination (N98, OR = 4.4 (3.2–5.9)), ovarian dysfunction (E28, OR = 1.5 (1.4–1.7)), endometriosis (N80, OR = 1.7 (1.5–2.0)), hydatidiform mole (O01, OR = 6.0 (3.8–9.5)), and three ICD codes related to the respiratory system—acute nasopharyngitis (J00), acute tonsillitis (J03), and acute bronchitis (J20). In total, 61 diagnosis codes with a significantly different prevalence in cases and controls were identified (Fig. 3, Supplementary Table S6).

## Discussion

We conducted the first GWAS meta-analysis for ectopic pregnancy in 7070 women with ectopic pregnancy and 248 810 female controls from two European-ancestry biobanks. We identify two genetic risk loci providing evidence of a genetic susceptibility component to this common early pregnancy complication. Our results inform the genetic background and provide clues to the etiopathogenesis of ectopic pregnancy.

We also characterize the phenotypic and genetic correlations with other phenotypes. The genetic correlation analyses provide further support for the association between ectopic pregnancy and smoking, which has been shown to be an important risk factor of ectopic pregnancy in past epidemiological studies (Saraiya *et al.*, 1998; Bouyer *et al.*, 2003; Horne *et al.*, 2014). A positive correlation with maternal smoking around birth may point towards a transgenerational effect on tubal function, similar to the one observed in the respiratory tract of offspring of smoking mothers (McEvoy and Spindel, 2017). However, caution is needed when interpreting this result since as far as we are aware, this is the first report of such an association and genetic correlations can be influenced by confounding factors. Phenotype-level analyses show increased prevalence of various reproductive health diagnoses in women with ectopic pregnancy, reflecting the previously known epidemiological associations between ectopic pregnancy and IVF, salpingitis, pelvic inflammatory disease, and others, and lending support to our ICD-code based phenotype definitions. While to some extent the increased prevalence of these disease codes in women with ectopic pregnancy is due to shared socio-economic and behavioural risk factors, it is unclear whether in some cases shared molecular mechanisms might also play a role. We also observe an increased prevalence of respiratory diagnoses related to sinusitis, tonsillitis, and bronchitis, which can all be linked to altered ciliary function, similar to ectopic pregnancy, since cilia in the respiratory tract epithelium help to clear mucus and protect against infections (Tilley *et al.*, 2015).

Recent years have seen numerous studies that have begun to clarify the genetic susceptibility to pregnancy complications such as pre-eclampsia (McGinnis *et al.*, 2017) or gestational diabetes (Pervjakova *et al.*, 2022). Less is known about the genetics of early pregnancy complications, which often are symptoms of unviable pregnancy and thus inevitably have a lower heritability. By combining data from two large population-based biobanks, we were able to reach a sample size that allowed us to identify the first genetic susceptibility factors for ectopic pregnancy and demonstrate a heritable component to the condition. Our research underlines the value of large-scale biobank resources to advance the study of pregnancy complications, of remarkable importance since these lack suitable animal models and there are ethical concerns on the use of human biopsies.

Our study provides genetic evidence supporting the role of *MUC1* in ectopic pregnancy. *MUC1* codes for a large epithelial apical surface glycoprotein that acts as a barrier to embryo implantation. Previous inconclusive small-scale studies have proposed that the expression of MUC1 in the Fallopian tubes of women diagnosed with ectopic pregnancy is reduced (Savaris *et al.*, 2008; Al-Azemi *et al.*, 2009; Refaat *et al.*, 2012). This has led to the hypothesis that altered expression of *MUC1* may predispose women to ectopic pregnancy. The results of the current study support this hypothesis, as the GWAS signal on chromosome 1 co-localizes with quantitative trait loci affecting both *MUC1* gene expression and specific transcript expression. Moreover, we were able to tie our association with a splice acceptor variant rs4072037, providing insight into the potential functional mechanisms underlying this association.

The T-allele (in previous literature referred to as the A allele) of rs4072037 results in a 27 bp/9 amino acid deletion in the 2nd exon of MUC1. Previous studies have shown that this may lead to changes in intracellular trafficking, glycosylation, and folding of the protein, which all may affect the function of MUC1, or alternatively, it has been shown that the same variant may also affect the transcriptional activity of the *MUC1* promoter (Saeki *et al.*, 2011). This variant is first and foremost known for its association with gastric cancer (Saeki *et al.*, 2011; Zhang *et al.*, 2011; Zheng *et al.*, 2013), and in this context, it is believed rs4072037 influences the quantity and/or the quality of the MUC1 protein. This causes a difference in its barrier function in the stomach and subsequently modifies the susceptibility to environmental risk factors that cause inflammation and carcinogenesis. Our genetic correlation results showing association between ectopic pregnancy and gastrointestinal diagnoses also reflect the shared genetic background between these two conditions. Altered barrier function of MUC1 in the Fallopian tubes is also a plausible explanation for its association with ectopic pregnancy. In line with this, rs4072037 has been associated with pregnancy loss and ectopic pregnancy also in the UK Biobank (Li *et al.*, 2021). However, due to the age structure of the UK Biobank, the results should be interpreted with caution since many of the actual cases are mislabelled as controls, resulting in extremely low prevalence of pregnancy phenotypes in this biobank cohort.

The association with the second locus on chromosome 10 is less clear and our GWAS follow-up analyses failed to provide any solid evidence to support the role of any specific gene. Given the low frequency of this variant (minor allele frequency < 1%), further replication studies are needed in populations where it is more common to confirm its association with ectopic pregnancy and propose potential explanations about the mechanisms.

While our study of more than 7000 cases identifies two genetic risk loci for ectopic pregnancy, further larger meta-analyses or independent studies are needed to validate these findings, especially regarding the rare variant on chromosome 10. Additionally, the limited available data from other ancestries hinders full assessment of the transferability of these findings across diverse populations. Moreover, as with other reproductive phenotypes, the lack of sufficiently sized relevant tissue data (in this case, Fallopian tube) in commonly used gene expression and other databases (such as the GTeX), hinders the proper interpretation of GWAS findings and highlights the need for large-scale gene expression studies in female reproductive tissues. Additional studies are also needed to evaluate whether smoking, an important risk factor of ectopic pregnancy, somehow mediates the genetic effects, along with studies evaluating additional risk factors and their interaction with ectopic pregnancy.

In conclusion, the first GWAS meta-analysis in ectopic pregnancy provides genetic evidence to support the involvement of the MUC1 epithelial glycoprotein and maps genetic and phenotypic associations with other phenotypes, providing input for further studies.

## Supplementary Material

dead217_Supplementary_Figure_S1

dead217_Supplementary_Figure_S2

dead217_Supplementary_Table_S1

dead217_Supplementary_Table_S2

dead217_Supplementary_Table_S3

dead217_Supplementary_Table_S4

dead217_Supplementary_Table_S5

dead217_Supplementary_Table_S6

dead217_Supplementary_Table_S7

## Data Availability

The GWAS summary statistics underlying this article are available in the GWAS Catalog, accession number GCST90272883.
