# Supplementary material for: Genome-wide association study meta-analysis supports association between MUC1 and ectopic pregnancy
Source: Hum Reprod. 2023 Oct 24;38(12):2516–25. doi: 10.1093/humrep/dead217 (PMC10694401; doi:10.1093/humrep/dead217)
Supplement: dead217_Supplementary_Figure_S1 [file dead217_supplementary_figure_s1.pdf]

Human Reproduction, 2023, 1–1
https://doi.org/10.1093/humrep/dead217
Supplementary Data

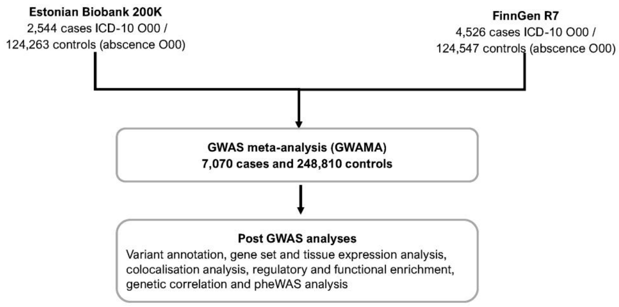

Supplementary Figure S1. Flowchart of the study design. ICD-10, International Classification of Disease 10; GWAS, genome-wide association study; pheWAS, phenome-wide association study.
