# Supplementary material for: Genome-wide association study meta-analysis supports association between MUC1 and ectopic pregnancy
Source: Hum Reprod. 2023 Oct 24;38(12):2516–25. doi: 10.1093/humrep/dead217 (PMC10694401; doi:10.1093/humrep/dead217)
Supplement: dead217_Supplementary_Figure_S2 [file dead217_supplementary_figure_s2.pdf]

OXFORD
human reproduction

Human Reproduction, 2023, 1–1
https://doi.org/10.1093/humrep/dead217
Supplementary Data

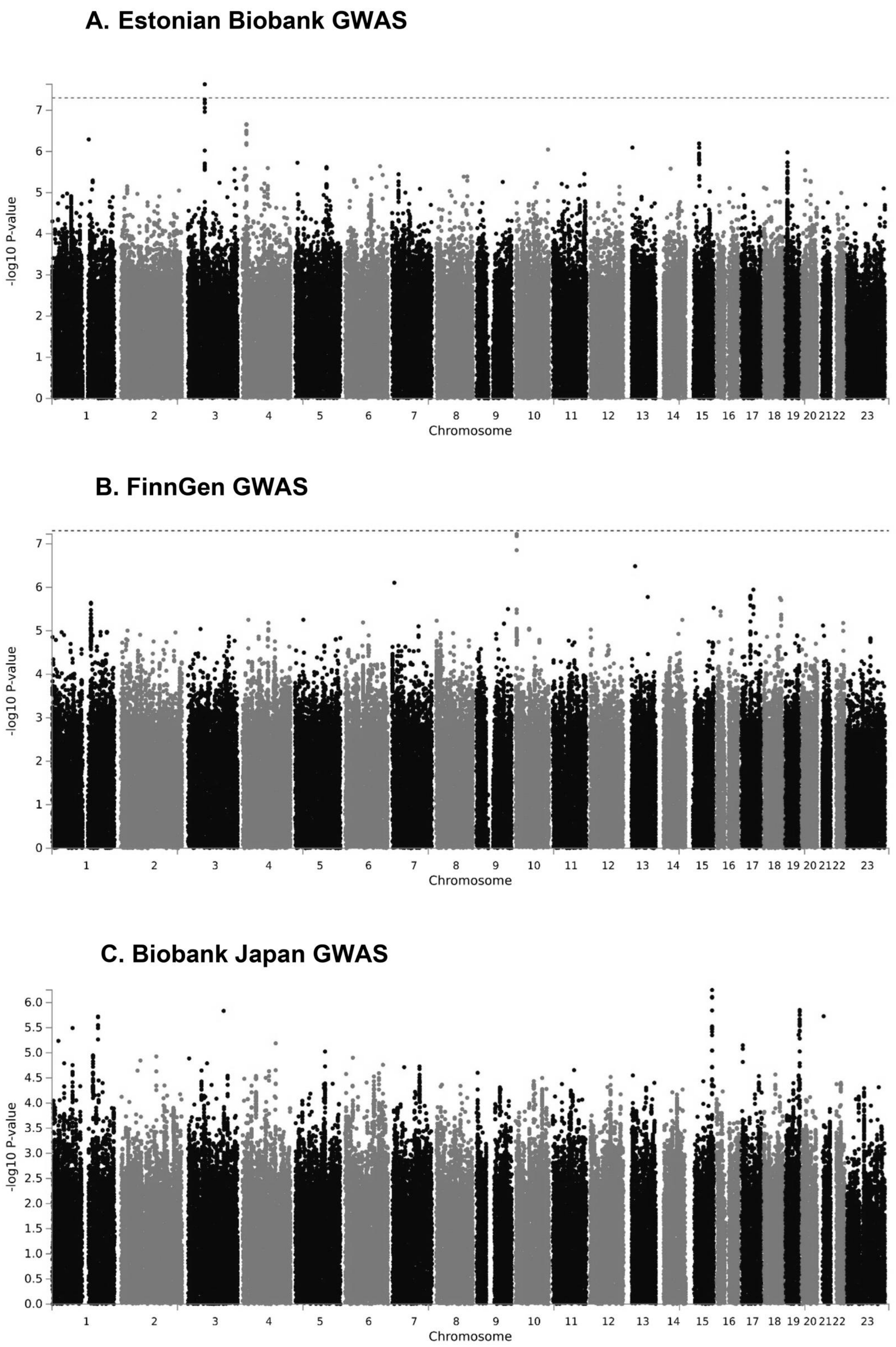

Supplementary Figure S2. Manhattan plots of cohort-level genome-wide association study (GWAS) for (A) Estonian Biobank, (B) FinnGen, and (C) Biobank Japan. On the Manhattan plot, the y axis represents –log10(*P*-values) for association of variants with ectopic pregnancy.
